# Analysis of Marketing Strategy for Food Supplements and Over-The-Counter Medicines

**DOI:** 10.3889/oamjms.2016.075

**Published:** 2016-07-04

**Authors:** Marjan Dzeparoski, Suzana Trajkovic-Jolevska

**Affiliations:** 1 *Bionika Pharmaceuticals Ltd., Skopje, Republic of Macedonia*; 2 *Faculty of Pharmacy, Ss. Cyril and Methodius University of Skopje, Skopje, Republic of Macedonia*

**Keywords:** marketing, marketing strategy, analysis, food supplements, over-the-counter medicines

## Abstract

Marketing strategy is correlated with the regulations for the corresponding product category. Accordingly, there is a big difference in the marketing strategy of food supplements and over-the-counter medicines. In this paper are presented 2 different marketing strategies of a new small pharmaceutical company in two studies. The findings of studies analysis can be used for developing marketing strategies in the wider sense and other products, for other small to medium sized companies in other countries of interest with similar regulations and help them understand how to position and promote themselves and their products.

## Introduction

Marketing strategy differs depending on the product, pharmaceutical company and market situation. The process consists of 3 phases: planning, implementation and control [1-3]. The strategy can relate to therapeutic groups, product line or individual product [4]. In this paper, 2 different marketing strategies of the new small pharmaceutical company in two studies will be presented. In Study One will be explored marketing strategy for food supplements (product line). In Study Two will be explored and created a marketing strategy for over-the-counter (OTC) medicine (product strategy) for a non-EU pharmaceutical company (Macedonia) to enter the EU market (Slovenia), based on the comprehensive research analysis. Today pharmaceutical companies must sell smarter and deliver greater value with smaller sales teams.

## Methods

In both studies are used qualitative and literature research. Additionally, in Study One are used questionnaires for wholesalers and pharmacies and competitors analysis. In Study Two are used situation analysis (Political, Economic, Regulatory, Technological, Social/PERTS & SWOT analysis) and interviews with company employees.

## Results

### Study One

Products are positioned as food supplements that support the health of patients. Starting points of the strategy are:

- Brand names of food supplements - easy to remember
- Visual differentiation through packaging – impressiveness
- Abstract display of elements/effects on packaging
- Modern and clear design - associated with modern sophisticated technology

The concept for the brand name is to mark the effect which food supplements have on the human body (some names are a blend of ingredients that are contained or the organ for which they are intended, others mark the effect).

On the packages is displayed modern graphic typography concept, letters contribute to playfulness, visual impressiveness and authenticity, complemented by body parts that affect the supplements. Organs are presented in an interesting cubist manner, by emphasising the tissue as a creator of every organ and this gives original minimalist outlook that sets it apart as unique packaging sample.

Communication objectives are: creating company awareness, promoting a line of food supplements, further education of target audience for their action and the way of usage.

Target audiences are professionals - doctors, pharmacists, associations and non-governmental organisations, and the general public - medias.

Limited launch phase (preparatory phase) is represented by testing the products with special target groups (physicians), building company credibility and presentation of products among experts.

Introduction phase of food supplements is represented by presenting the company and announcement of products – public relations, launch, positioning, point of sale and point of purchase support, creating awareness (cooperation with portals that have headings for health) through mass electronic media - TV and print media campaigns for the professionals and general public.

#### PR activities:

1. Presentation of the company - company brand through an introduction to general public and specific audiences, by demonstrating capacity and technology through interviews with selected media.

Objective: presentation to the public and target audiences, promoting investment and highlighting the contribution of the company to the economy and quality of life.

Target audience: representatives of medias, companies (partners, suppliers, customers), experts, general public.

2. Building credibility - strengthening the company position by initiating important topics related to contemporary trends in company’s interest at target audiences.

3. Products presentation - communication-related to certain products and advice for their positive characteristics using “third party”.

Objective: to inform the public about products advantages and characteristics, promotion, strengthening confidence in products recommended by physicians and pharmacists.

Target audience: the general public.

According to the presented data, it is obvious that registration of food supplements is not demanding as the registration process of medicines, so their replacement with other food supplements is also easier and predicted. Usually, they are hit products which have a shorter life cycle, about 4-5 years, which is not the case with the medicines. Requirements in terms of investments in the manufacturing facilities, equipment and staff are also much smaller in terms of requests for necessary good pharmaceutical practices that are imperative for the pharmaceutical product manufacturers. Manufacturers of food supplements need only an HACCP certificate. Food supplements pricing is free and is not regulated by law.

### Study Two

Marketing plan for the OTC product is developed based on the tabulated data from situation analysis - PERTS analysis (Table 1) and SWOT analysis (Table 2).

**Table 1:** PERTS analysis – macro environment

External factors	Opportunities	Threats
**Political**	Reimbursement list and health care policies are subject to change	Removal from reimbursement list

**Economic**	Economical situation is improving Medicine market grows	Economic crisis Change of exchange rate

**Regulatory**	Obtaining marketing authorization	Regulatory changes

**Technological**	Growth by expanding production with semisolid and liquid pharmaceutical dosage forms, medical devices and cosmetics	Competition New innovative competitors technology

**Social**	Direct-to-Consumer pharmaceutical advertising Good relationships with pharmacists and physicians	Limited economic opportunities Dependence on personal relationships with pharmacists and physicians Consumer brand affection

**Table 2:** SWOT analysis – micro & macro environment

**STRENGTHS**	**WEAKNESSES**
Experienced staff Trained sales representatives Reputation for quality	Competition is well established and employees are well educated Limited resources Not so innovative products

**OPPORTUNITIES**	**THREATS**
Expansion of product portfolio Medicine market grows Reimbursement list and health care policies are subject to change Economical situation is improving	Reduction of prices of competitors products Decreased interest among distributors due to competitive products Decreased interest among key practitioners Removal from reimbursement list

PERTS analysis model is exploring the external factors, particularly useful for international markets.

SWOT analysis is an analysis of both, internal and external company factors (Table 2).

Based on two presented analyses, we found weak and critical points of the marketing plan and process optimisation of the medicine launch (ML) is proposed. Optimisation process of ML is important to eventually reduce or exclude certain risks that can cause failure or poor results. They would have a lasting negative effect on the positioning and the image of the company and products in markets where growth in sales is expected.

#### Critical points

There are more activities and key points which are important for the smooth development of ML, which may cause difficulties or halt the process if not monitored carefully. Involved are the finance department, education, legal resources, registration & pharmacovigilance, quality control, production, research & development, procurement & marketing. Some of the activities are well organised, but some need to be organised better:

1. Marketing/Management

Communication is imperative, is very important between pharmaceutical company from Macedonia and the Slovenian partner responsible for the marketing of OTC medicine, as well as managing the employees in each of the companies. Employees should know to whom to address for certain issues. Also, the perception of Slovenian market is important, because although there are similarities, there are social and cultural differences between Macedonia and Slovenia that influence decision-making [5].

- *Project leader*. Important is to appoint the project leader who will allocate tasks, monitor the activities and implementation of timelines and budget frames. This will facilitate to get answers quickly and to know always in which phase is ML.

- *Goals*. Goals motivate employees to achieve them. Goals should be realistic and achievable, in order not to cause opposite effect among employees, but also should not be too low. They should be measurable and specific for certain time period.

- *Communication*. It is defined in the plan for ML and consists of meeting for ML, limited launch phase (LLP), local promotion of medicine, full launch phase (FLP) and choice of marketing materials.

- *Sales representatives*. OTC medicine is scheduled to be sold through sales representatives, as well as direct marketing and advertising for the general public. Success is depending on whether sales representatives are motivated enough.

- *Traceability and communication*. It is very important for the team to be willing to achieve good results. It is necessary to monitor and communicate the success or failure, to know at what stage ML is and whether earlier stages were successful.

- *Price*. Price formation is complex and depends on regulation, international markets and expectations of the company. Pricing of OTC medicines in Macedonia is free, but for example in Slovenia [6] and in other countries price is regulated by law.

- *LLP centres*. Sales representatives should select key physicians and centres that will be included in the limited launch phase. The country manager should decide which of the selected centres are crucial and will support OTC medicine.

- *Marketing materials*. Once marketing sectors agreed on the selection of promotional materials, the materials will be translated into Slovenian. Slovenian partner has full responsibility for this activity.

- *Organising events*. Marketing team participates in events organisation by selecting hotels, transport, advertising materials and other activities or hires PR and travel agencies.

2. Education

Training level depends on the importance of ML. Education will be carried out internally at the pharmaceutical company and externally in the business partner premises.

*Internal education* is the education of sales representatives and includes presentations for OTC medicine and therapy, and marketing training, as well. For all companies, it is important to have experienced sales representatives who are continually educated. Training should be interesting and useful for the participants.

*External education* is the education of the local partner sales representatives. The external educational program will be provided for Slovenian physicians and pharmacists, as well. This way, the awareness for OTC medicine will be created. Participants will acquire information about the way and duration of use, packaging, and will have the opportunity to test the medicine.

3. Legislation/Regulation

*Laws*: legal sector should check earlier legal requirements for contracts with distributors, their validity and possibility of automatic extending. The legal sector could explore the possibility for certain exclusivity because the law on anti-monopoly behaviour for medicines does not allow exclusivity.

*Regulation*: EU GMP certificate is a prerequisite for obtaining medicines marketing authorization in EU. Without this certificate, it is not possible medicines to be released in EU, also in some non-EU countries which have implemented EU legislation [7-9]. The process of obtaining EU-GMP certificate is complex, laborious and expensive, requires great employee’s efforts and lasts minimum one year.

Propositions for optimising ML

- *Communication and employee management*. Managing staff and communication with the responsible employee is very important for companies; especially in ML. Communication can be improved by determining contact responsible person for each part. The person manages the flow of communications and forwards them to the appropriate person. This way the performance and speed are improved. PR agency can be engaged, committed to publishing texts for OTC medicine, ML and the timetable for the launch. Changing this way, communication is better and employee involvement is greater.

- *Creating campaign*. It is very important to create a campaign for a certain problem that can be cured through the use of OTC medicine. The use of medicine can be maximised, where it has not been used before or it has been used to a lesser extent (an indication for which the medicine has not been developed originally, but for which the medicine offers an appropriate solution) [10]. Awareness can be created through meetings or interviews with physicians, pharmacists, consumers, sales representatives, and through direct marketing and advertising to the general public. This will create customers satisfaction, offering a solution to their needs.

- *Internal delays*. Internal delays may be caused by differences in priorities of both partners and include late decision making, timelines are not always respected, the last moment meeting cancellations. Problems can be overcome if more independence is given to the Slovenian business partner in decision making or by involving more company employees in the activities.

*- Marketing materials*. Different type of promotional materials can be used in marketing. In this case, it is important ML, therefore appropriate materials for ML have to be chosen. It is important because not all marketing materials have the same importance in different approaches of marketing strategies. Marketing materials should be ordered and delivered on time.

*- Production/Procurement, Distribution*. Although forecasts and orders can be given in time, there may be delays in transportation/delivery of the product (e.g., delay in clearance), problems can arise in production, delay in delivery of any material which enters the manufacturing process of medicine, leading to time prolongation in planned deliveries. One solution could be storing larger stock of necessary raw materials, which requires more storage space and brings higher costs. A better solution would be closely monitoring of all activities and full involvement by the responsible persons from the company. This would make possible more objective and effective product distribution (certain product quantities for specific region) based on the regional sales data.

Because of the size of investment and expectations, the approach in marketing strategy development is much more serious, more complex and broader for OTC medicines. The presented data show that LLP and FLP reference centers are selected very carefully, critical points are set and monitored, proposals for optimization of ML are given, in order to avoid any marketing strategy delay or failure, which would have long lasting negative effect on the positioning and image of the pharmaceutical company and products in markets where sales growth is expected. As the product is OTC medicine, the approach must be supported with much more scientific data and therefore patients and healthcare professionals take them very seriously.

In conclusion, marketing strategy is correlated with the regulations for the corresponding product category. Registration of food supplements is easier and much quicker than registration of medicines. As a result, there is a big difference in the marketing strategy of food supplements and OTC medicines. Studied findings can be used for developing marketing strategies in the wider sense and other products, for other small to medium sized companies in other countries of interest with similar regulations.
